# A Novel, Easy Assay Method for Human Cysteine Sulfinic Acid Decarboxylase

**DOI:** 10.3390/life11050438

**Published:** 2021-05-14

**Authors:** Angela Tramonti, Roberto Contestabile, Rita Florio, Caterina Nardella, Anna Barile, Martino L. Di Salvo

**Affiliations:** 1 Istituto di Biologia e Patologia Molecolari, Consiglio Nazionale delle Ricerche, Piazzale Aldo Moro 5, 00185 Roma, Italy; angela.tramonti@cnr.it (A.T.); anna.barile@uniroma1.it (A.B.); 2 Istituto Pasteur Italia–Fondazione Cenci Bolognetti, Dipartimento di Scienze Biochimiche “A. Rossi Fanelli”, Sapienza Università di Roma, P.le A. Moro, 5, 00185 Roma, Italy; roberto.contestabile@uniroma1.it (R.C.); caterina.nardella@uniroma1.it (C.N.); 3 European Brain Research Institute, Fondazione “Rita Levi-Montalcini”, 00185 Roma, Italy; r.florio@ebri.it

**Keywords:** cysteine sulfinic acid decarboxylase, pyridoxal 5′-phosphate, circular dichroism, enzymatic assay

## Abstract

Cysteine sulfinic acid decarboxylase catalyzes the last step of taurine biosynthesis in mammals, and belongs to the fold type I superfamily of pyridoxal-5′-phosphate (PLP)-dependent enzymes. Taurine (2-aminoethanesulfonic acid) is the most abundant free amino acid in animal tissues; it is highly present in liver, kidney, muscle, and brain, and plays numerous biological and physiological roles. Despite the importance of taurine in human health, human cysteine sulfinic acid decarboxylase has been poorly characterized at the biochemical level, although its three-dimensional structure has been solved. In the present work, we have recombinantly expressed and purified human cysteine sulfinic acid decarboxylase, and applied a simple spectroscopic direct method based on circular dichroism to measure its enzymatic activity. This method gives a significant advantage in terms of simplicity and reduction of execution time with respect to previously used assays, and will facilitate future studies on the catalytic mechanism of the enzyme. We determined the kinetic constants using L-cysteine sulfinic acid as substrate, and also showed that human cysteine sulfinic acid decarboxylase is capable to catalyze the decarboxylation—besides its natural substrates L-cysteine sulfinic acid and L-cysteic acid—of L-aspartate and L-glutamate, although with much lower efficiency.

## 1. Introduction

Taurine (2-aminoethanesulfonic acid) is the most abundant free amino acid in animal tissues and is highly present in liver, kidney, muscle, and brain, accounting for 25%, 50%, 53%, and 19% of their free amino acid pools, respectively [1]. Many works have shown that taurine plays numerous biological and physiological roles [2]. In fact, not only does it conjugate bile acids [3], but it also plays a role as antioxidant [4,5], osmoregulator [6], and membrane stabilizer [7]. In the central nervous system, taurine acts as neurotransmitter, neuroprotective agent, and regulator of intracellular calcium homeostasis [8]. Low levels of taurine are associated with various pathological conditions, including cardiomyopathy [9], retinal degeneration [10], prenatal and postnatal growth retardation [11], and obesity [12]. Thanks to its beneficial properties on human health, taurine is added in most infant milks [13], in energy drinks, and in cosmetics [14].

**Figure 1:**
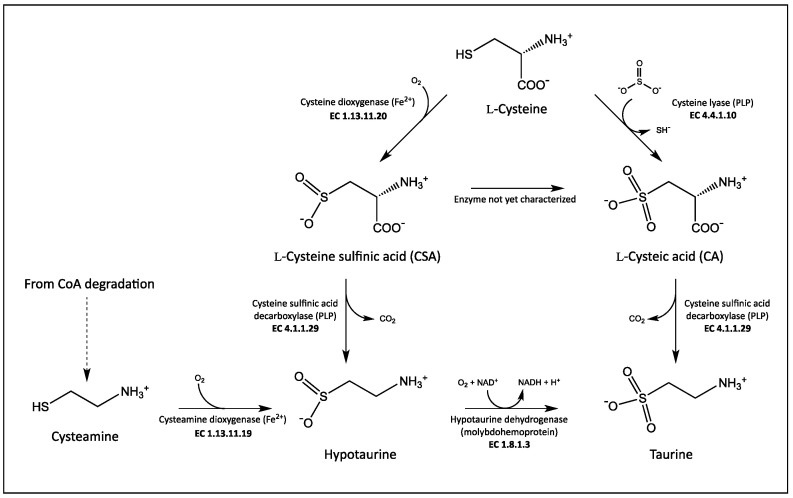
Taurine biosynthetic pathway in mammals.

Taurine derives from L-cysteine (Figure 1). The major route for its synthesis is through the decarboxylation of L-cysteine sulfinic acid (CSA) to hypotaurine by cysteine sulfinic acid decarboxylase (CSAD; EC 4.1.1.29), and the subsequent oxidation of hypotaurine to taurine. An alternative pathway is the oxidation of CSA to L-cysteic acid (CA), followed by the decarboxylation of CA to taurine catalyzed by a decarboxylase that is to be CSAD as well [15]. Despite the importance of taurine in human health, human CSAD has been poorly characterized at the biochemical level. It belongs to the aspartate aminotransferase fold type I superfamily of pyridoxal-5′-phosphate (PLP)-dependent enzymes [16]. Significantly, several other PLP-dependent enzymes are involved in taurine biosynthesis (Figure 1). CSAD is expressed in brain and liver from two tissue-specific mRNAs that differ in their 5′-untranslated regions due to an alternative splicing, yet result in two identical proteins [17]. In vivo and in vitro studies suggested that CSAD activity increases when the enzyme is phosphorylated, and it is inhibited when dephosphorylated, with protein kinase C and protein phosphatase 2C probably involved in this regulation [18]. Among PLP-dependent decarboxylases, CSAD shows the strongest homology with the two isoforms of glutamate decarboxylase, GAD65 and GAD67 [17], responsible for the decarboxylation of L-glutamate to produce γ-aminobutyrate (GABA), the main inhibitory neurotransmitter of the central nervous system. It has been shown that GAD is able to decarboxylate both CSA and CA [15], whereas no activity of CSAD was found with L-glutamate [17]. The substrate selectivity of GAD and CSAD is determined by the identity of amino acids occupying three specific positions at the active site of the two enzymes [19]. A taurine biosynthetic pathway has also been found in bacteria, and CSAD from *Synechococcus* sp. PCC 7335 was recombinantly expressed and purified [17]. On the other hand, taurine seems not to be present in plants, although a novel biosynthetic pathway rising from L-serine was quite recently identified in microalgae [20].

In the present work, we have recombinantly expressed and purified human CSAD, and applied a simple spectroscopic, direct method to measure its enzyme activity with CSA and CA. This method gives a significant advantage in terms of simplicity and reduction of execution time, with respect to previously used assays such as that based on *o*-phthalaldehyde derivatization of products and high-performance liquid chromatography detection [19]. We also showed that human CSAD is capable to catalyze the decarboxylation of L-aspartate and L-glutamate, although with much lower efficiency.

## 2. Materials and Methods

### 2.1. Materials

Ingredients for bacterial growth, chemicals, and enzyme substrates (CSA, CA, L-glutamate, and L-aspartate) in pure form were purchased from Sigma-Aldrich (St. Louis, MO, USA). HisTrap affinity columns (Ni-NTA) for purification of the 6xHis-tagged protein were purchased from GE Healthcare (Chicago, IL, USA).

### 2.2. Purification of CSAD

The plasmid pNIC28-Bsa4 containing the cDNA of CSAD (UniProt entry code Q9Y600) was a gift from Nicola Burgess-Brown (Addgene plasmid # 42387). It is derived from the pET28a vector. In this vector, the cDNA of CSAD (GenBank: AAH98342.1) is fused to an N-terminal tag of 23 residues (MHHHHHHSSGVDLGTENLYFQS) including a hexahistidine (His6) and a TEV-protease cleavage site. The plasmid was transformed into *E. coli* Rosetta(DE3) cells, containing pLys helper plasmid.

An overnight culture of these cells was used to inoculate 2 L of Vogel-Bonner medium E containing kanamycin (40 mg·L^−1^), chloramphenicol (35 mg·L^−1^), and pyridoxine (30 mg·L^−1^). Bacteria were allowed to grow for approximately 5 h at 37 °C (until their OD_600_ reached ~0.6), then the growing temperature was lowered to 25 °C and the expression of CSAD was induced with 0.1 mM isopropyl thio-β-D-galactopyranoside (IPTG). Bacteria were harvested after 18 h of growth at 25 °C and suspended in 50 mL of 100 mM NaHEPES, pH 8, containing 500 mM NaCl, 5% glycerol, 0.2 mM dithiothreitol (DTT), and an ethylenediaminetetraacetic acid (EDTA)-free protease inhibitor (Sigma-Aldrich). Cell lysis was carried out by sonication on ice (5 min in short 10 s pulses, with 5 s intervals). Lysate was centrifuged at 12,000× *g* for 30 min to remove insoluble debris. The supernatant was loaded onto a column of Ni-NTA agarose resin pre-equilibrated with buffer A (20 mM NaHEPES, pH 7.5, containing 500 mM NaCl, 5% glycerol, and 0.2 mM DTT). The column was washed with 10 volumes of buffer A, 10 volumes of buffer A containing 10 mM imidazole, and eluted with buffer A containing 500 mM imidazole. Pooled fractions containing CSAD, as detected by SDS-PAGE analysis, were concentrated and washed in 20 mM NaHEPES, pH 7.5, containing 300 mM NaCl, 5% glycerol, and 0.2 mM DTT, using centrifuge ultrafiltration devices (Sartorius).

### 2.3. Spectroscopic Measurements

All spectroscopic measurements were carried out at 20 °C in 20 mM NaHEPES buffer, pH 7.5, containing 300 mM NaCl, 0.2 mM DTT, and 5% glycerol. UV-visible spectra were recorded with a Hewlett-Packard 8453 diode-array spectrophotometer (Agilent Technologies). The growth of bacterial cultures was monitored by determining the optical density at 600 nm (OD_600_), using the same diode array spectrophotometer. Protein subunit (molecular weight 57.576 kDa) concentration was calculated using an extinction coefficient at 280 nm of 63,576 M^−1^ cm^−1^ (calculated with the Gill and Von Hippel method; [21]). Circular dichroism measurements were carried out using a Jasco 710 spectropolarimeter. Acquisition parameters were as follows: start 250 nm–end 190 nm; data pitch: 0.5 nm; scanning speed: 10 nm/min, response: 0.25 s; bandwidth: 2 nm; accumulation: 3.

### 2.4. Thin Layer Chromatography

Samples of 2 μL of reaction mixtures were spotted on silica plates (Merck) with CSA, CA, glutamate, aspartate, and β-alanine as standards. The mobile phase was a mixture of butanol:acetic acid:H_2_O in the 3:1:1 ratio. After chromatography, amino acids were revealed upon treatment with 0.5% ninhydrin in acetone, followed by heating at 90 °C.

### 2.5. Activity Assay of CSAD with CSA and CA

The activity of CSAD versus CSA and CA was assayed by circular dichroism (CD) measurements. Decarboxylation of CSA and CA was followed by measuring the decrease of the CD signal at 220 nm due to the conversion of these L-amino acids into an achiral amine (taurine and hypotaurine, respectively). Far-UV (190–250 nm) CD spectra of CSA and other potential amino acid substrates (CA, L-glutamate, and L-aspartate), measured using a Jasco 710 spectropolarimeter equipped with a DP 520 processor and a 1 cm path length quartz cuvette, are shown in Figure 3b. All amino acids show a positive CD band, although of different intensity. CSAD has a negative CD band that does not interfere with the measurement since the enzyme concentration keeps constant during the assay. The reactions were started by adding 0.5 mM CSA or 2 mM CA to a solution of 0.65 μM CSAD in 20 mM potassium phosphate buffer, pH 7.2, containing 0.2 mM DTT, 0.1 mM EDTA, and 0.06 mM PLP. The decrease of CD signal at 220 nm was monitored over time. This wavelength was chosen so as to maximize the signal, while maintaining a linear relationship between CD signal and amino acid substrate concentration. The reaction rate was expressed as variation of ellipticity per minute (mdeg min^−1^).

### 2.6. Activity Assay of CSAD with Glutamate

The reaction of CSAD with L-glutamate was assayed using GABase (Sigma-Aldrich, St. Louis, Mo, USA), a commercial preparation containing GABA aminotransferase and succinic semialdehyde dehydrogenase, which is normally employed to measure GAD activity [22]. CSAD (30 μM) was incubated at 30 °C with 50 mM sodium L-glutamate in 20 mM potassium phosphate buffer, pH 7.2, containing 0.2 mM DTT, 0.1 mM EDTA, and 0.6 mM PLP. Aliquots of this reaction mixture were taken at time intervals (5–90 min) and halted by boiling for 5 min. After centrifugation, each aliquot (50 μL) was mixed with 0.2 mL of GABase solution (0.1 M NaHEPPS, pH 8.6, containing 1 mM NADP^+^, 1 mM α-ketoglutarate, 3 mM mercaptoethanol, and 0.6 unit/mL of GABase), pre-activated at room temperature for 20 min. After 40 min at 37 °C, the reaction mixture was diluted to 1 mL with water, and the amount of NADPH formed—which is equimolar to the GABA produced—was determined by measuring the absorbance at 340 nm using an extinction coefficient of 6220 M^−1^cm^−1^.

### 2.7. Data Analysis

All data analyses were carried out using the software Prism (GraphPad Software Inc., San Diego, CA, USA). Steady-state kinetic parameters were obtained by nonlinear least squares fitting of initial velocity data to the Michaelis–Menten equation.

## 3. Results

### 3.1. Expression, Purification, and Spectrophotometric Analysis of Human CSAD

**Figure 2:**
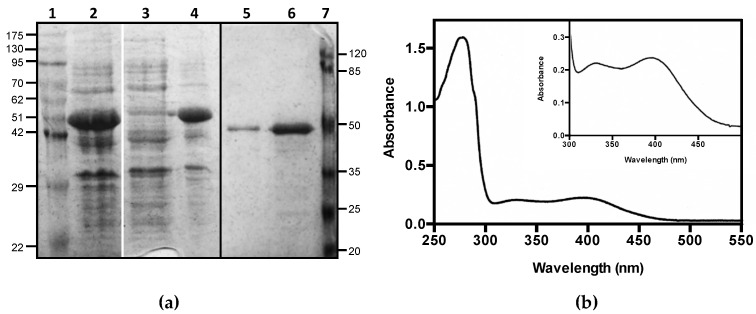
Purification of CSAD. (**a**) SDS-PAGE analysis of total protein extract (2); soluble (3) and insoluble (4) fractions of lysate; 1 μg (4) and 5 μg (5) of purified human CSAD. Molecular weight standards are indicated in kDa (lane 1 and 7). The black line indicates that the image was assembled from two different gels, whereas the white line separates different parts of the same gel that were combined, excluding lanes that were not of interest; (**b**) Absorption spectrum of purified human CSAD. UV–VIS absorption spectrum was recorded in 20 mM NaHEPES buffer pH 7.5, containing 300 mM NaCl, 0.2 mM DTT, and 5% glycerol.

Human CSAD (EC 4.1.1.29), whose cDNA was cloned in pNIC28-Bsa4, was expressed in the *E. coli* Rosetta(DE3) strain with an N-terminal tag of 23 residues including a hexa-histidine stretch. The recombinant protein was purified to homogeneity, as judged by SDS-PAGE analysis (Figure 2a, original figure is in Supplementary Materials). Although most of the expressed protein was found in the inclusion bodies (lane 4 vs. 3 in Figure 2a), the purification yield was of about 3 mg of soluble protein per liter of bacterial culture. The absorption spectrum of the purified CSAD suggested that only a very small amount of PLP was bound to the protein. The addition of exogenous PLP—in equimolar concentration with respect to the protein—restored the holo-form of the enzyme, as demonstrated by the measurement of a neat increase in catalytic activity (see below for details on the assay method). After extensive dialysis, the presence in the absorption spectrum of two absorption bands at 330 and at 400 nm (Figure 2b) suggested that PLP was retained by the protein, although absorption bands with maxima at these wavelengths usually correspond to free PLP [23]. However, aldimines absorbing between 385 and 400 nm have been reported in the literature in different PLP-dependent decarboxylases [24,25,26]. In order to keep the protein in the holo-form, excess PLP was added to the purified protein sample and to all reaction mixtures during activity assays. In these conditions, the protein was stable for several weeks at 4 °C.

### 3.2. Activity Assay: Decarboxylation of CSA and CA

**Figure 3:**
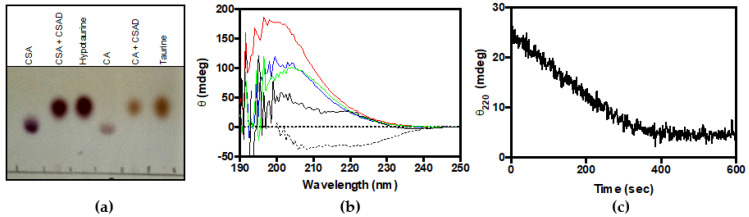
Decarboxylation of CSA and CA. (**a**) Thin-layer chromatography of reaction Scheme 10. (mM) with CSAD (30 μM) at 30 °C for 1 h in 50 mM potassium phosphate buffer, pH 7.5, containing 0.2 mM DTT, 0.1 mM EDTA, and 0.08 mM PLP. Samples of CSA, CA, hypotaurine, and taurine were run as references; (**b**) CD spectra of 0.5 mM CSA (black line), CA (red line), L-glutamate (blue line), and L-aspartate (green line). As control, the CD spectrum of 3 µM CSAD (dotted line) was also recorded; (**c**) The decarboxylation reaction of CSA was monitored as change of ellipticity at 220 nm as a function of time. The CD signal was measured as a function of time upon mixing CSAD (0.66 μM) with 0.5 mM CSA in 20 mM potassium phosphate buffer, pH 7.2, containing 0.2 mM DTT, 0.1 mM EDTA, and 0.06 mM PLP at room temperature.

**Figure 4:**
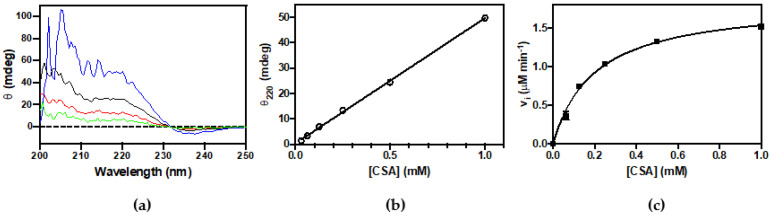
Determination of kinetic parameter for CSA decarboxylation by circular dichroism. (**a**) CD spectra of CSA at 0.125 mM (green), 0.25 mM (red), 0.5 mM (black), and 1 mM (blue); (**b**) Dependence of ellipticity (θ) at 220 nm as a function of CSA concentration. The θ was measured at different concentration of CSA. The fitting of data with a linear regression gives a value of 50 ± 0.7 mdeg per 1 mM of CSA; (**c**) Ellipticity at 220 was measured as a function of time upon mixing CSAD (0.33 μM) with CSA (0.06–1 mM) in 20 mM potassium phosphate buffer, pH 7.2, containing 0.2 mM DTT, 0.1 mM EDTA, and 0.06 mM PLP. The continuous line through the experimental points is the result of least squares fitting of data to the Michaelis–Menten equation, which yielded estimated k_cat_ and K_M_ values of 5.6 ± 0.2 s^−1^ and 0.2 ± 0.02 mM, respectively.

The activity of CSAD was first checked by thin-layer chromatography analysis. From Figure 3a, it is clear that the purified enzyme is able to use both CSA and CA as substrates, converting them to hypotaurine and taurine, respectively. These results demonstrate that recombinant human CSAD is active. In order to quantitatively assay the decarboxylase activity of CSAD, we set up a method based on CD measurements. This method has been previously used to determine the activity of other decarboxylases such as ornithine decarboxylase [27] and diaminopimelate decarboxylase [28]. It is based on the fact that amino acid substrates have a chiral center (the α-carbon) and show a far-UV CD spectrum (Figure 3b), whereas their decarboxylation products do not. Therefore, as the irreversible decarboxylation reaction proceeds, the CD signal decreases as a result of the conversion of the amino acid into an achiral amine. As shown in Figure 3c, the complete decarboxylation of 0.5 mM CSA (into hypotaurine), monitored at 220 nm in the presence of 0.66 μM CSAD at 20 °C, was accomplished in less than 400 s. We also tested 3 mM CA, L-glutamate, and L-aspartate, but we observed activity only with CSA (−0.080 mdeg min^−1^ per μM of CSAD) and CA (−0.0045 mdeg min^−1^ per μM of CSAD). Possibly, with this method and under such conditions, the reaction rates of L-glutamate and L-aspartate decarboxylation were too low to be measured. The kinetics parameters of CSA decarboxylation were determined. In order to convert the observed ellipticity changes into the concentration of CSA consumed in the decarboxylation reaction, a calibration curve was constructed, relating CSA concentration to CD signal. CD spectra of CSA at concentrations ranging from 0.125 to 1 mM are shown in Figure 4a. Figure 4b shows that the CD signal at 220 nm was linear up to 1 mM CSA concentration. The initial rate of CSA decarboxylation was measured as a function of CSA concentration, obtaining a saturation curve from which a k_cat_ of 5.6 ± 0.2 s^−1^ and a K_M_ equal to 0.20 ± 0.02 mM were determined (Figure 4c).

### 3.3. Decarboxylation of L-Aspartate and L-Glutamate

**Figure 5:**
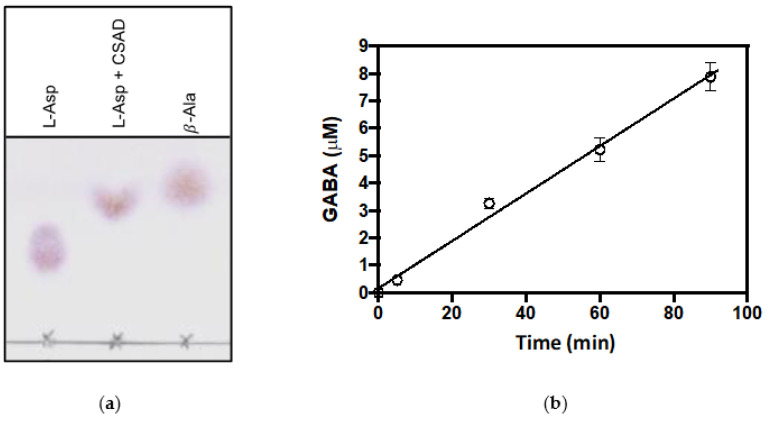
Decarboxylation of L-aspartate and L-glutamate. (**a**) Thin-layer chromatography of reaction samples obtained by incubation of L-aspartate (mM) with CSAD (30 μM) at room temperature overnight in 20 mM potassium phosphate buffer, pH 7.2, containing 0.2 mM DTT, 0.1 mM EDTA, and 0.08 mM PLP. Samples of L-aspartate and β-alanine were run as references; (**b**) Determination of GABA formation in the reaction of 32 μM CSAD with 50 mM sodium glutamate, in 20 mM potassium phosphate buffer, pH 7.2, containing 0.2 mM DTT and 0.1 mM EDTA. Measurements of amounts of GABA were carried out using the GABase assay, as described in Materials and Methods. Linear regression of data gave a velocity value of 0.09 μM·min^−1^.

CSAD-catalyzed decarboxylation of L-aspartate was observed by thin-layer chromatography analysis after a prolonged incubation of a 10 mM solution of this amino acid with 30 μM of CSAD, at 20 °C for 16 h (Figure 5a). Decarboxylation of L-glutamate could instead be monitored using the GABase assay, as explained in the Material and Methods section. Using this assay, 30 μM of CSAD was incubated with 50 mM sodium L-glutamate at 20 °C, and the product GABA was measured at time intervals (Figure 5b). These results clearly show that CSAD is able to decarboxylate L-glutamate, with a rate of 0.003 μM·min^−1^ per μM of CSAD, but is also able to slowly decarboxylate L-aspartate.

## 4. Discussion

Human CSAD with an N-terminal hexahistidine tag could be expressed very well in the *E. coli* Rosetta(DE3) strain, although most of the protein was insoluble. By growing bacteria in minimal medium at low temperature we were able to purify up to 3 mg of soluble enzyme per liter of bacterial culture (Figure 2). The enzyme was active towards different substrates, such as CSA, CA, L-aspartate, and L-glutamate (Figure 3, Figure 4 and Figure 5). CSAD activity with CSA as substrate was assayed by other authors using different methods, such as a radioisotope assay that uses L-[1-^14^C]CSA and measures the formation of ^14^CO_2_ [15], and the measurement of the hypotaurine reaction product, derivatized with *o*-phthalaldehyde and detected by high-performance liquid chromatography [17,19,29]. The CD assay method is simple and direct, because it does not require any step after the decarboxylation reaction. It has been applied to other enzymes [27,28,30], but it has never been used for CSAD. By applying this method, we determined the kinetics constants of CSA decarboxylation (Figure 4). The K_M_ value of 0.2 mM found for human CSAD is very similar to the K_M_ measured for the enzyme extracted from bovine brain [15]. Moreover, the k_cat_ value of 5.6 s^−1^ is similar to that observed with other human decarboxylases, such as GAD (6–7 s^−1^; [31]) and DOPA decarboxylase (3–9 s^−1^; [32]). Our method allowed us to demonstrate that human CSAD can also decarboxylate CA, although at a slower rate with respect to CSA (−0.0045·mdeg min^−1^ vs. −0.08 mdeg min^−1^). This result is in line with the hypothesis that CSAD is also responsible of the decarboxylation of CA to taurine in the alternative pathway of taurine biosynthesis (Figure 1; [15]).

In contrast to what was previously reported in literature, namely, that CSAD is unable to decarboxylate L-glutamate [17], we observed a slow reaction also with L-glutamate and L-aspartate. The decarboxylation of L-glutamate is rather expected, because CSAD is highly homologous to GAD [17], which, on the other hand, can decarboxylate CSAD substrates, CSA and CA [15,31,33].

**Figure 6:**
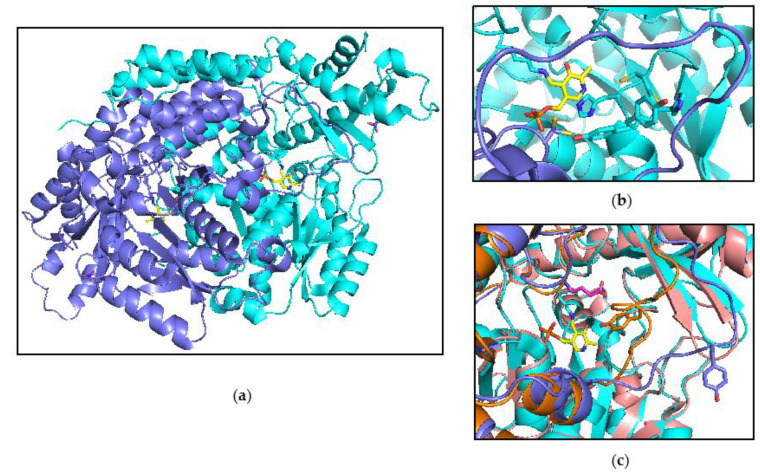
Structure of CSAD. (**a**) Overall structure of the dimeric form of the enzyme. The protein backbone is depicted as cartoon. Chain A and chain B are shown in cyan and slate, respectively. The PLP (in yellow color) bound to the active site residue Lys305, and the nitrate are shown as sticks. (**b**) Close-up of the active site loop comprising residues 190–192. The loop was modelled in two different conformations. Residues Cys190, His191, Tyr192, and Cys356 are shown as sticks; (**c**) Comparison between the putative catalytic loop of CSAD—comprising residues Ser331 to Lys341—with the structurally related loop of human GAD67 (PDB 2okj). The catalytically relevant tyrosine residues present on this loop are shown as sticks. Chain A and chain B of CSAD are shown in cyan and slate, respectively. Chain A and chain B of GAD 67 are shown in salmon and orange. For clarity, only PLP bound to CSAD is shown, yellow sticks. GABA bound to GAD67 is shown as pink sticks.

The comparison of the catalytic features of human CSAD with its paralogs human glutamate decarboxylases (GAD65 and GAD67) and DOPA decarboxylase (DDC) might take advantage of the three-dimensional structure of CSAD, which has been solved to a resolution of 1.6 Å by the Structural Genomics Consortium (SGC) (PDB accession code 2jis). The overall structure of human CSAD is shown in Figure 6a. The enzyme crystallized as a homo-dimer with the PLP cofactor bound through a Schiff base to lysine 305 in both monomers. A nitrate molecule is clearly seen in the electron density, coordinated to His431, Trp415, Gly462, and Arg461; this nitrate molecule may stabilize the loop containing the latter two amino acids. The active site cavity is formed by both monomers. Within both active sites, the loop containing Cys190/His191/Tyr192 can be modelled in two alternative conformations. In the “closed” conformation, the His191 at the center of this loop coordinates with the PLP. Cys356 is also found in two conformations, possibly influenced by the proximity of His191 (Figure 6b). An important structural feature of PLP-dependent decarboxylases is a putative catalytic loop that covers the active site. Part of this loop, from residue Ser331 to Lys341, is unstructured in chain A, while in chain B it is modelled in a much more open conformation when compared to the structurally and functionally similar GAD67 (Figure 6c). This overall relaxed conformation of the active site may reflect the absence of a bound substrate or product molecule in the CSAD structure. In GAD67, this loop has been proposed to bring catalytically important residues into the proximity of PLP and substrate [34]; for instance, a highly conserved tyrosine residue present in this loop is responsible for substrate and reaction specificity in human DOPA decarboxylase [35].

The novel activity assay described in the present work will help site-directed mutagenesis investigations on the structural bases of substrate specificity and catalytic mechanism of human cysteine sulfinic acid decarboxylase.

## Data Availability

The three-dimensional structure of human CSAD can be found in the PDB databank (accession number 2jis).

## Supplementary Materials

The following are available online at https://www.mdpi.com/article/10.3390/life11050438/s1, Figure S1: SDS-PAGE gels. Lane numbering shown in Figure S1 is the same shown in Figure 2a. The lanes that are not part of Figure 2a are red-crossed in Figure S1.
