# Supplementary material for: A Novel, Easy Assay Method for Human Cysteine Sulfinic Acid Decarboxylase
Source: Life (Basel). 2021 May 14;11(5):438. doi: 10.3390/life11050438 (PMC8153620; doi:10.3390/life11050438)
Supplement: Supplementary file 1 [file life-11-00438-s001.zip › Supplementary Materials REVISED DI SALVO-ori-done.pdf]

Supplementary Materials

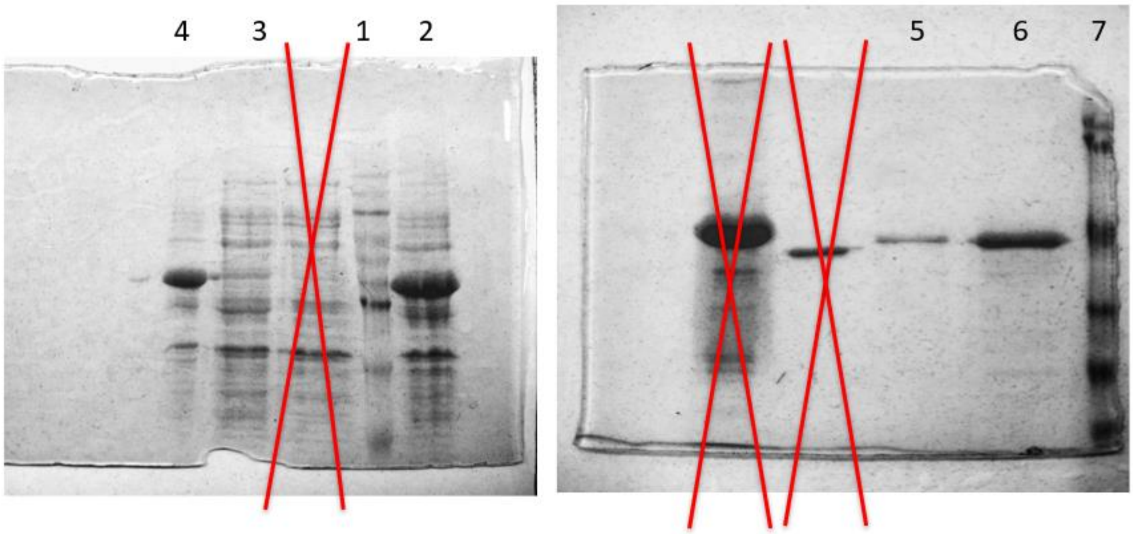

Figure S1. SDS-PAGE gels. Lane numbering shown in these figures is the same shown in Figure 2a. The lanes that are not part of Figure 2a are red-crossed.
